# Supplementary material for: Meta-Analysis of the Effects of Biochar Application on the Diversity of Soil Bacteria and Fungi
Source: Microorganisms. 2023 Mar 2;11(3):641. doi: 10.3390/microorganisms11030641 (PMC10057247; doi:10.3390/microorganisms11030641)
Supplement: Supplementary file 1 [file microorganisms-11-00641-s001.zip › microorganisms-2238083-supplementary.pdf]

Meta-Analysis of the Effects of Biochar Application on the Diversity of Soil Bacteria and Fungi

Mingyu Wang [1], Xiaoying Yu [1], Xiaohong Weng [1], Xiannan Zeng [2], Mengsha Li [3,*] and Xin Sui [1,*]

1 Engineering Research Center of Agricultural Microbiology Technology, Ministry of Education & Heilongjiang Provincial Key Laboratory of Ecological Restoration and Resource Utilization for Cold Region & Key Laboratory of Microbiology, College of Heilongjiang Province & School of Life Sciences, Heilongjiang University, Harbin 150080, China

2 Institute of Crop Cultivation and Tillage, Heilongjiang Academy of Agricultural Sciences, Harbin 150088, China

3 Institute of Nature and Ecology, Heilongjiang Academy of Sciences, Harbin 150040, China

* Correspondence: lms19861004@163.com (M.L.); xinsui_cool@126.com (X.S.)

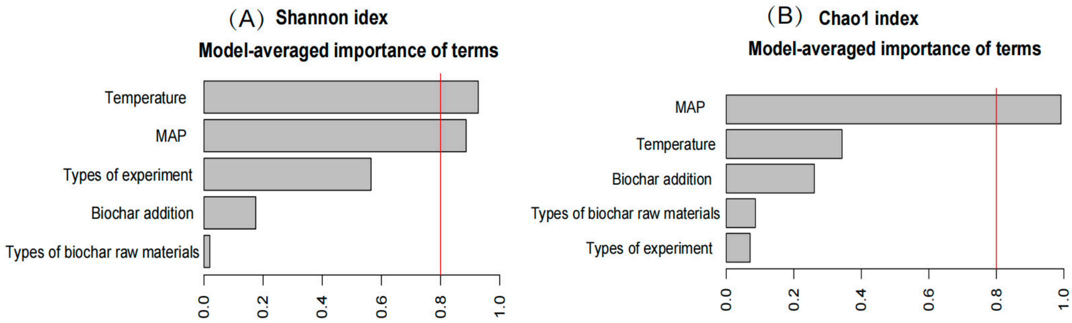

Figure S1. Multifactorial importance analysis of the bacterial Shannon index (A) and the bacterial Chao1 index (B).

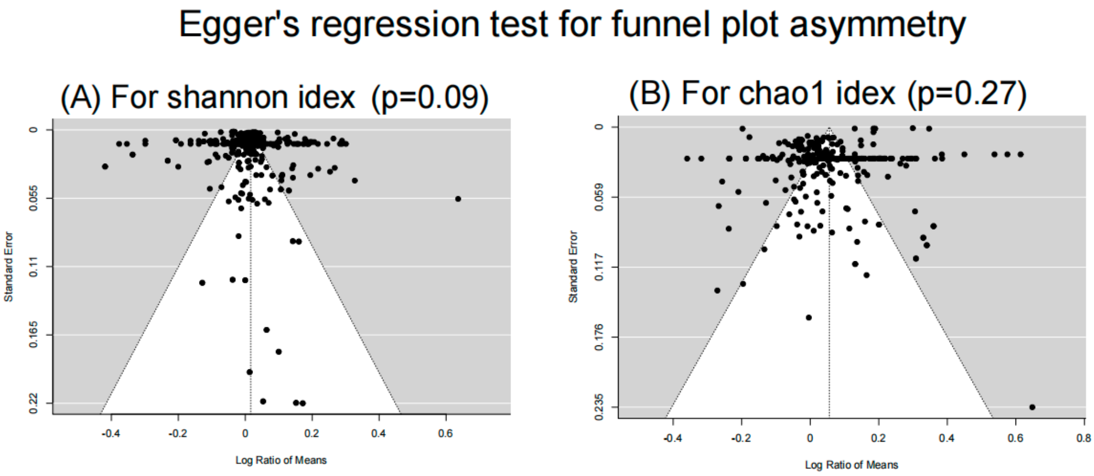

Figure S2. Egger's regression test for funnel plot asymmetry. (A). Soil bacteria shannon index. (B). Soil bacteria chao1 idex. When the model p-value is greater than 0.05 ($p > 0.05$) it means that the funnel shape is symmetrical and the results are less affected by publication favorability.
